# A chimeric influenza virus vaccine expressing fusion protein epitopes induces protection from human metapneumovirus challenge in mice

**DOI:** 10.3389/fmicb.2022.1012873

**Published:** 2023-12-14

**Authors:** Tian Chongyu, Lei Guanglin, Sun Fang, Deng Zhuoya, Yang Hao, Li Cong, Li Xinyu, He Wei, Tan Lingyun, Niu Yan, Yang Penghui

**Affiliations:** ^1^Fifth Medical Center of Chinese PLA General Hospital, Beijing, China; ^2^College of Animal Science and Veterinary Medicine, Shanxi Agricultural University, Taigu, China; ^3^Department of Immunology, School of Basic Medical Sciences, Anhui Medical University, Hefei, China; ^4^Inner Mongolia Medical University, Hohhot, China; ^5^First Medical Center of Chinese PLA General Hospital, Beijing, China

**Keywords:** influenza virus, RG technology, HMPV, fusion protein, HMPV vaccine

## Abstract

Human metapneumovirus (HMPV) is a common virus associated with acute respiratory distress syndrome in pediatric patients. There are no HMPV vaccines or therapeutics that have been approved for prevention or treatment. In this study, we constructed a novel recombinant influenza virus carrying partial HMPV fusion protein (HMPV-F), termed rFLU-HMPV/F-NS, utilizing reverse genetics, which contained (HMPV-F) in the background of NS segments of influenza virus A/PuertoRico/8/34(PR8). The morphological characteristics of rFLU-HMPV/F-NS were consistent with the wild-type flu virus. Additionally, immunofluorescence results showed that fusion proteins in the chimeric rFLU-HMPV/F-NS could work well, and the virus could be stably passaged in SPF chicken embryos. Furthermore, intranasal immunization with rFLU-HMPV/F-NS in BALB/c mice induced robust humoral, mucosal and Th1-type dominant cellular immune responses *in vivo*. More importantly, we discovered that rFLU-HMPV/F-NS afforded significant protective efficacy against the wild-type HMPV and influenza virus challenge, with significantly attenuated pathological changes and reduced viral titers in the lung tissues of immunized mice. Collectively, these findings demonstrated that chimeric recombinant rFLU-HMPV/F-NS as a promising HMPV candidate vaccine has potentials for the development of HMPV vaccine.

## Highlights

- We generated a chimeric recombinant influenza virus vector vaccine carrying partial HMPV-F proteins.
- The morphological characteristic of rFLU-HMPV/F-NS was consistent with wt flu virus, and it could stably passage in SPF chicken embryos.
- rFLU-HMPV/F-NS could elicit robust humoral, mucosal, and Th1-type dominant cellular immune responses *in vivo*, thereby providing effective protection against wt HMPV challenge in mice.
- These findings highlight the chimeric recombinant virus rFLU-HMPV/F-NS has potential as a promising HMPV vaccine.

## Introduction

Human metapneumovirus (HMPV), first isolated in 2001, is a newly identified respiratory pathogen of the pneumothoracic family (Van den Hoogen et al., 2001; Feuillet et al., 2012; Panda et al., 2014). HMPV is the primary cause of approximately 20% of deaths under 5 years of age and threatens the health of over 86% of children under 5 years of age in the world (Walker et al., 2013; Céspedes et al., 2016; Perchetti et al., 2020). The clinical symptoms of HMPV infection were cough, fever, runny nose, wheezing, and dyspnea (Lefebvre et al., 2016). No specific therapy or vaccine for HMPV is currently available to date. Therefore, there is an urgent need to develop safe and effective anti-HMPV strategies.

HMPV encodes nine different proteins from eight genes; both the fusion (F) and attachment (G) genes produce surface glycoproteins (Perchetti et al., 2020). The F protein induces neutralizing antibodies and is broadly genetically stable, and most antibodies against HMPV are directed against the F protein (Uche and Guerrero, 2018). Notably, the F protein has been shown to bind to integrins that act as cellular receptor, which take part in the attachment and fusion of the hMPV particle to target cells at the respiratory tract (Van den Hoogen et al., 2004; Cox et al., 2012). Herein, the F protein is essential for viral entry and is a crucial target for neutralizing antibodies and vaccine development.

Reverse genetic (RG) techniques based on influenza A virus (IAV) are well established and can be cultured and produced in chicken embryos and cell cultures. Mouse and ferret models have successfully assessed the immunoprotective effects of recombinant IAV (de Goede et al., 2009; Jones et al., 2011; Li et al., 2013). The coding sequence of NS1 can accommodate large segments of non-IAV sequences that induce host-specific immune responses by expressing exogenous antigens (Stukova et al., 2006). Respiratory viruses such as recombinant bovine parainfluenza virus (PIV) PIV3, PIV1 and Sendai virus have been tested as vector systems for the expression of the HMPV F protein (Uche and Guerrero, 2018; Russell Charles and Hurwitz, 2021). Virus-like particles(VLPS), bacterial vectors and other systems can also induce the expression of helper T cells and cytotoxic memory T cells and promotes clearance of HMPV from mouse lungs by expressing the HMPV F protein (Mok et al., 2008; Talaat et al., 2013; Palavecino et al., 2014; Bates et al., 2016). However, no IAV reverse genetics-based HMPV vaccines have been constructed and reported.

We have previously developed a recombinant influenza virus rFLU/RSV/F + G, which carries a human syncytial virus (RSV) epitope on the backbone of influenza virus A/PR/8/34, eliciting strong protective immunity (Chengrong et al., 2014). In this report, we inserted the first 909 nt of the HMPV-F coding region into the NS gene fragment of IAV (H1N1)PR8, termed rFLU-HMPV/F-NS. We evaluated the immunogenicity and protective efficacy of immunization with rFLU-HMPV/F-NS in a mouse model of HMPV infection.

## Materials and methods

### Cells lines and viruses

COS I cells, MDCK cells, and Vero-E6 cells were purchased from the Chinese Academy of Sciences; these cells were cultured in Dulbecco’s modified Eagle’s medium (DMEM; Gibco, Grand Island, NY, United States) containing 10% fetal bovine serum. The use of the cell lines was approved by the Ethics Committee of the Fifth Medical Center of Chinese PLA General Hospital. The wild-type influenza virus A/PR/8/34(PR8), A/China/BJMY011/2020(H1N1) were grown in 9–11 day-old specific pathogen-free (SPF) chicken embryos (Beijing Laboratory Animal Center, China). HMPV-BJ1812 was grown in Vero-E6.

### Rescue of recombinant influenza virus engineered to express the HMPV-F protein

Recombinant viruses carrying the HMPV F protein were produced using influenza virus reverse genetics technology. In brief, the coding sequences of the HMPV F protein were downloaded from GenBank. The full-length sequences of NS1 were optimized and synthesized by Sangon Biotech (Shanghai). The first 909 nt of the HMPV F protein-coding region insert into the non-structural protein NS1 gene fragment of influenza virus A/PR/8/34 (PR8). The bidirectional expression vector pHW2000 was used to clone the pFLU-HMPV/F-NS. After sequencing, the recombinant confirmed plasmids and the other seven plasmids encoding influenza PR8 virus(pHW-PB2, pHW-PB1, pHW-PA, pHW-HA, pHW-NP, pHW-NA, and pHW-M) were co-transfected into MDCK and COS I cells (ratio 1:2) by Effectene Transfection Reagent (Qiagen, Hilden, Germany). The chimeric virus rFLU-HMPV/F-NS was harvested when a hemagglutination-positive result was observed with the HA assay. The titers of the rFLU-HMPV/F-NS virus were determined with a 50% tissue culture infectious dose (TCID_50_) in MDCK cells.

### Electron microscopy assay

The recombinant virus rFLU-HMPV/F-NS was concentrated by ultracentrifugation and purified in a 30 and 60% sucrose density gradient. The purified virus rFLU-HMPV/F-NS was obtained by deglycosylation. After negative staining, the morphology and size distribution of the recombinant virus rFLU-HMPV/F-NS were observed by transmission electron microscopy and compared with the typical structure of influenza viruses.

### Virus growth curve

The replication ability of rFLU-HMPV/F-NS was examined on MDCK cells at different time points. The supernatants of 12, 24, 48, 72, and 96 h cells were collected, and the virus titers were measured at the indicated time point.

### Transmission stability of recombinant virus rFLU-HMPV/F-NS

The recombinant virus rFLU-HMPV/F-NS chicken embryo allantoic fluid was collected and inoculated with 9-day-old SPF chicken embryos for passaging, blinded for five generations, and hemagglutination test and TCID_50_ assay were performed in each generation.

Haemagglutination test was determined by 96-well plates with 2-fold serial dilution of recombinant virus. 1% chicken red blood cells add to each well for 30 min at room temperature, and results were observed and recorded.

TCID_50_ titer of recombinant virus rFLU-HMPV/F-NS was determined by 96-well cell plates with 2 × 10^5^/mL MDCK cells. The recombinant virus rFLU-HMPV/F-NS was diluted 10-fold with serum-free DMEM infected cells for 5 days, and the cell supernatant with hemagglutination plate was determined by 1% chicken erythrocytes. Calculate the TCID_50_ for each generation of recombinant virus by Reed-Muench method (Neumann and Kawaoka, 2001).

### Immunofluorescence assay

The purified third-generation recombinant virus rFLU-HMPV/F-NS infected MDCK cells cultured on 24-well plates(200 μl/well) for 24 h, resulting in an infection multiple (MOI) of 1, along with negative control and PR8 virus control, 4% formaldehyde was fixed for 30 min; 1% TritonX-100 was permeabilised for 15 min; 5% BSA was blocked for 30 min; 1% BSA diluted primary antibody (Influenza A virus Nucleoprotein antibody, Anti-Metapneumovirus Fusion protein antibody) diluted 50-fold and incubated overnight at 4°C; 1% BSA 1:100 diluted fluorescent secondary antibody [Goat Anti-Rabbit IgG H&L antibody (Alexa Fluor®488), Goat Anti-Mouse IgG antibody (DyLight594)] for 1 h in the dark; DAPI staining for 5–10 min; the cell crawls were placed on the anti-fluorescence quencher and observed under an OLYMPUS confocal microscope.

### Animal experiments

All animal experiments were performed in accordance with the Institutional Animal Care and Use Committee and Ethics Committee of the Fifth Medical Center of Chinese PLA General Hospital. All facilities were approved by the Animal Care and Ethics Committee of the Fifth Medical Center of Chinese PLA General Hospital.

Seventy-two 6-week-old female BALB/c mice were randomly divided into three groups, rFLU-HMPV/F-NS virus 10^4^TCID_50_ dose group (low dose group, 21 mice), rFLU-HMPV/F-NS virus 10^6^TCID_50_ dose group (high dose group, 21 mice) and PBS control group (30 mice). BALB/c mice were anesthetized with isoflurane before immunization and immunized I.N. at 30 μl/each; the booster immunization was performed on day 28 after the prime immunization. Blood was collected from the fundus vein of each mouse as negative serum before immunization; before the booster immunization, blood was collected from the fundus vein of 3 mice from each group, and day 14 after the booster immunization, three mice from each group had their eyes removed for blood collection and lung lavage fluid and mouse spleen tissue were collected.

### Hemagglutinin inhibition assays

Serum samples were mixed with RDE (cholera bacteriocin) at a ratio of 1:4 and incubated at 37°C for 16–18 h. Samples were heated in a water bath at 56°C for 50 min and stored at 4°C. To determine the HA potency of A/China/BJMY011/2020 (H1N1), virus samples were serially diluted to 4 HA units and assayed on HA inhibition reaction plates for 1 h at room temperature. HA inhibition potencies in immunized mice were analyzed using GraphPad Prism 6.0 statistical software.

### Neutralization assays

Sera were diluted with cell culture maintenance solution by multiple dilution, 1:10; 1:40; 1:160; 1:640. Diluted antibody was mixed 1:1 with HMPV virus(100 MOI) and incubated for 1 h at 37°C. The incubated virus-serum mixture was added VERO-E6 well and incubated at 37°C for 2 h. Cells were cultured in complete media for 7 days at 37°C. On day 7, 100 μl of supernatants were collected and analyzed *via* qRT-PCR. Ct values were recorded and the potency of anti-HMPV neutralizing antibodies in immunized mice was calculated using the Reed-Muench method.

### Serum anti-HMPV IgG titers

1% BSA diluted serum, 1:200; 1:400; 1:800; 1:1,600 up to 1:102,400; HMPV virus coated ELISA plate, 100 μl/well, overnight at 4°C; blocking solution (3% BSA) blocked for 90 min; diluted serum incubate for 1 h; dilute HRP-labeled sheep anti-mouse IgG with 1% BSA at 4000-fold dilution, incubate for 1 h; 2 M H_2_SO_4_ to terminate the color development; The absorbance values of the reaction wells were measured by Synergy H4 ELISA at 450 nm, results were analyzed using GraphPad Prism 6.0 statistical software.

### Mucosal anti-HMPV sIgA titers

As above, the primary antibody was lung lavage solution with 1% BSA to dilute the sample from 2-fold ratio; the secondary antibody was HRP-labeled sheep anti-mouse sIgA at 4,000-fold dilution with 1% BSA.

### Flow cytometric assay

Day 14 after the booster immunization, three mouse spleens were taken from each group, separated and diluted to 2 × 10^5^/ml splenic lymphocyte suspension with 5 ml mouse splenic lymphocyte isolate; stimulating solution (200 μl saline + 0.5 μl stimulating agent + 15.87 μl HMPV), stimulated at 37°C for 6 h. CD4-FITC extracellular antibodies incubated cells in dark for 30 min at 37°C; fixative solution labeled cells for 20 min in dark; intracellular antibodies against TNF-α (APC), IFN-γ (PE), IL-2 (BV421), IL-4 (PE/CY7) labeled for 40 min in dark; washed with 1 × PB and centrifuge at 8000 rmp for 1 min, resuspended cell precipitate with 1 × PB, pended BD Flow cytometry assay.

### Wild-type virus challenge

All groups were infected with 15 LD_50_/ml A/China/BJMY011/2020 (H1N1) and 10^6^ PFU/mL HMPV-BJ1812 *via* nasal drip(30 μl/mice) at 2 weeks after the booster immunization in two groups per group (The PBS group was divided into three groups), under isoflurane anesthesia. The mice were observed for changes in body weight and the presence or absence of respiratory symptoms. On day 4 after influenza virus infection and day 6 after HMPV infection, three mice from each group were executed and left lung tissue was taken for viral load determination and right lung tissue was taken for pathological examination. The survival rate and weight change of the other 6 mice in each group were observed for 14 days.

### Viral titers

Influenza virus load in lung tissues were detected by TCID_50_ assay. Three mice of A/China/BJMY011/2020 (H1N1) strain challenge group were dissected after alcohol disinfection, and left lung tissues were extracted, ground by adding DMEM medium, centrifuged, and the supernatant was filtered through a 0.2 μm bacterial filter to remove bacteria, and the filtrate was collected for virus TCID_50_ assay.

HMPV viral load in mice lung tissues were assayed by Q-PCR method, and primers for HMPV F gene and mouse GAPDH gene were designed and synthesized by Shanghai Biotech. HMPV F Forward-Primer: 5′-CTTTGGACTTAATGACAGATG-3′; Reverse-Primer: 5′-GTCTCCCTGTGCTAACTTTG-3′.

GAPDH Forward-Primer: 5′-AGGTCGGTGTGAACGGATTTG-3′; Reverse-Primer: 5′-TGTAGACCATGTAGTTGAGGTCA-3′ Q-PCR was performed using the PowerUp™ SYBR™ Green Master Mix kit. The conditions were: 95°C for 10 min, 95°C for 15 s, 60°C for 60 s, 40 cycles, and 72°C for 30 s.

### Pathology

On day 4 post-A/China/BJMY011/2020 (H1N1) challenge and day 6 post-HMPV challenge, the right lungs of the mice was removed and fixed in 4% formaldehyde solution. The lungs of immunized mice were isolated, fixed, dehydrated, embedded in paraffin. HE staining was used to observe peribronchiolar inflammation, perivascular inflammation and alveolar inflammation.

### Statistical analysis

All statistical analyses were performed with GraphPad Prism v9.0.0 (GraphPad Software, CA, United States). Student’s t-test was used to analyze the differences between two groups. Analysis of variance (ANOVA) was performed to compare multiple groups. Two-sided *p* < 0.05 was defined as statistically significant.

## Results

### Generation of rFLU-HMPV/F-NS

**Figure 1:**
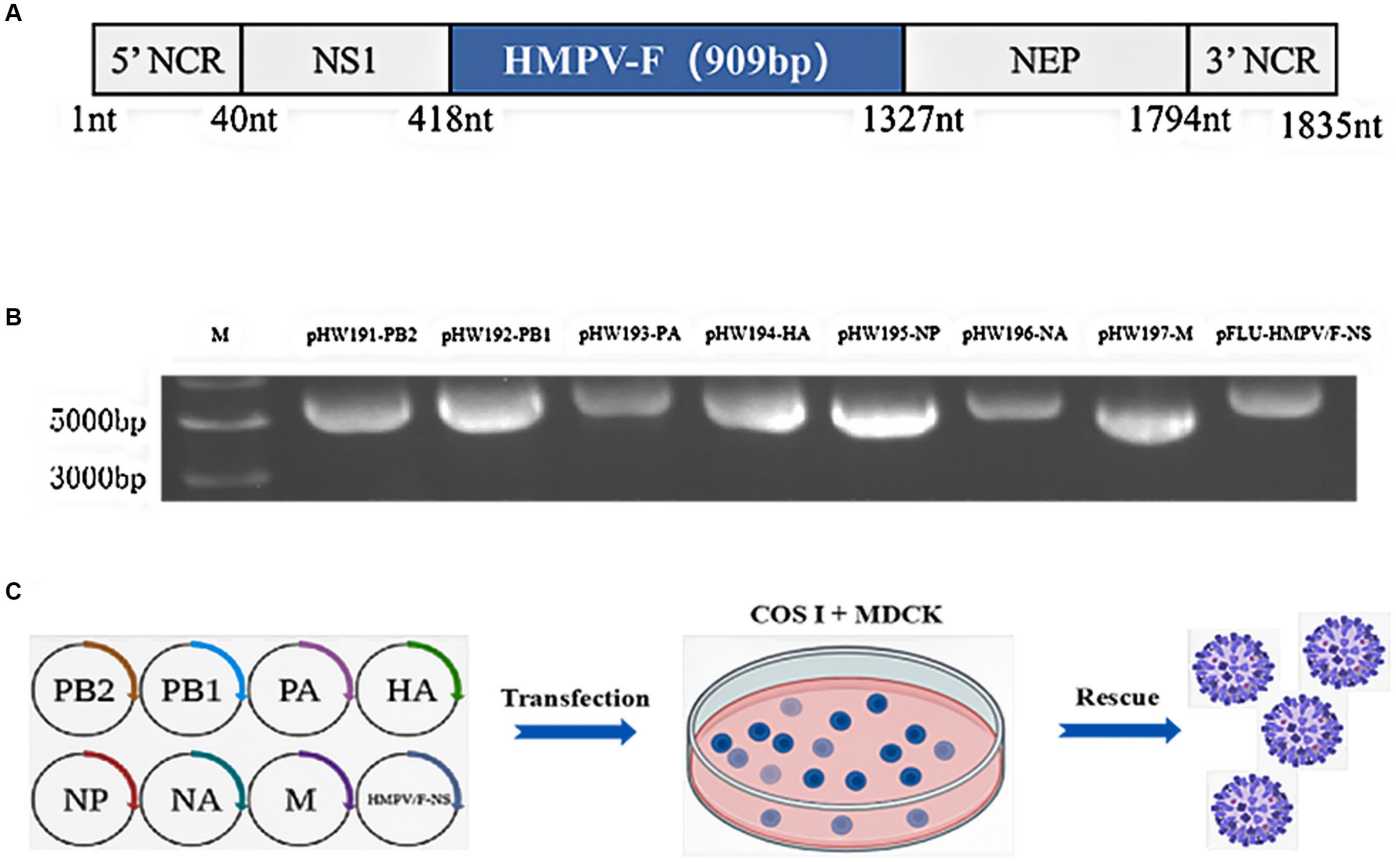
Construction and characterization of pFLU-HMPV/F-NS. **(A)** Construction strategy of plasmid pFLU-HMPV/F-NS; **(B)** Detection of transgenic segments in pFLU-HMPV/F-NS. M: Trans15K DNA Marker; **(C)** Schematic representation of the IAV reverse genetics system used for the production of rFLU-HMPV/F-NS. MDCK and COS I cells were co-transfected with pHW-PB2, pHW-PB1, pHW-PA, pHW-HA, pHW-NP, pHW-NA, and pHW-M (ratio 1:2), using Effectene. rFLU-HMPV/F-NS was harvested from cell supernatants upon confirmation of HA positivity. Titers of rFLU-HMPV/F-NS were determined by TCID_50_.

In order to rescue the recombinant influenza virus carrying partial HMPV F protein, we inserted 1–909 nucleotides of the coding region of HMPV F protein into the fragment of the non-structural protein NS1 gene of PR8 virus (Figure 1A), and constructed the recombinant plasmid pFLU-HMPV/F-NS on pHW2000 vector by bioinformatics prediction and sequence optimization, which Biotech commissioned. The recombinant plasmids pFLU-HMPV/F-NS were successfully constructed with the sizes of 1,835 bp (Figure 1B). The recombinant plasmids pFLU-HMPV/F-NS and the other seven backbone plasmids pHW191-PB2, pHW192-PB1, pHW193-PA, pHW194-HA, pHW195-NP, pHW196-NA, and pHW197-M of PR8 were transfected into cocultured cells of COS I and MDCK by RG (Figure 1C). Rescued recombinant viruses were named rFLU-HMPV/F-NS.

### Characterizations of rFLU-HMPV/F-NS

**Figure 2:**
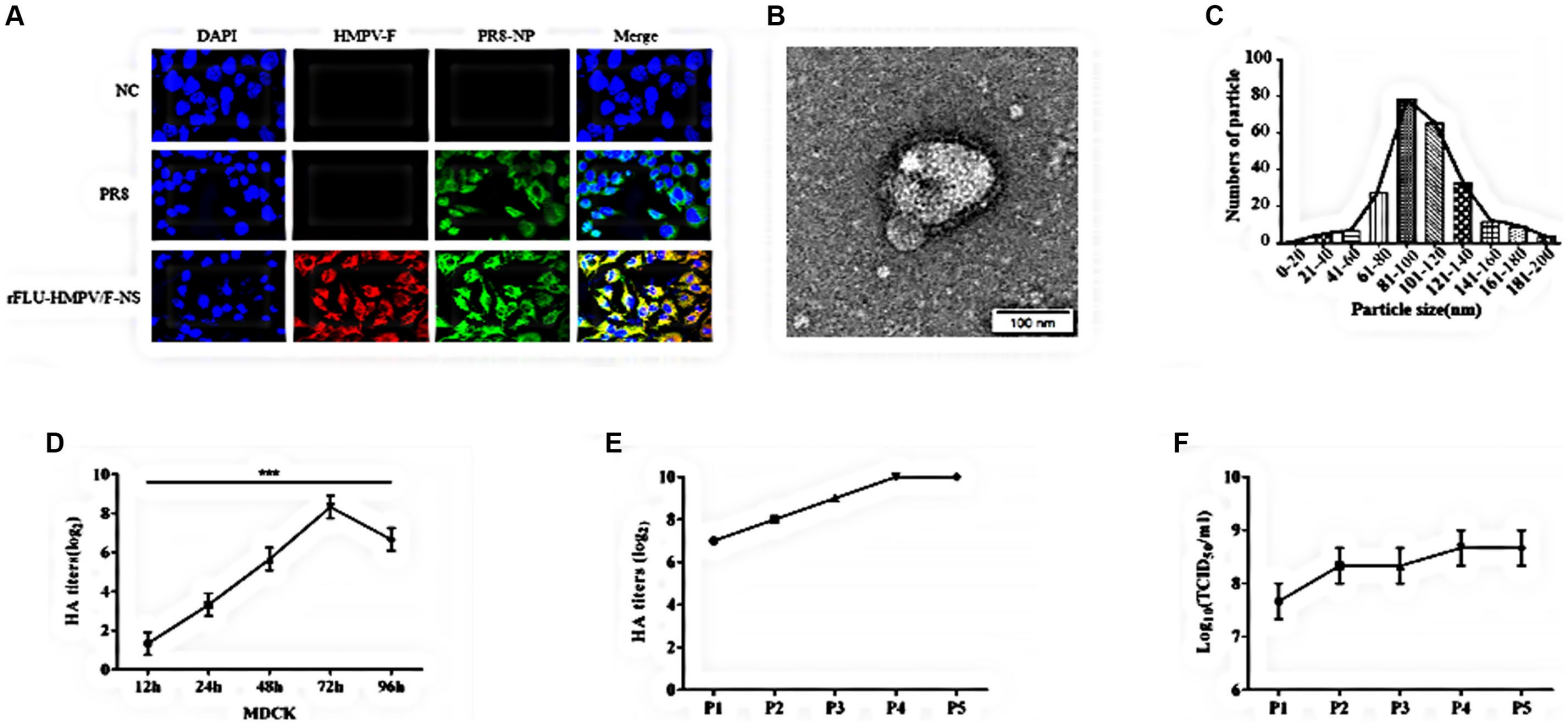
Identification of recombinant virus rFLU-HMPV/F-NS. **(A)** Immunofluorescent analysis of IVA and HMPV-F protein expression (60×). MDCK cells were infected for 24 h with purified 3rd generation rFLU-HMPV/F-NS (MOI: 1) and labeled with primary anti-IAV NP and anti-HMPV F antibodies. Cells were washed and stained with goat anti-rabbit IgG H&L (Alexa Fluor®488) and Goat Anti-Mouse IgG (DyLight594) for 1 h. Cells were imaged on an OLYMPUS confocal microscope. **(B)** Morphology and **(C)** size distribution of rFLU-HMPV/F-NS analyzed *via* TEM. **(D)** Growth curve of the recombinant virus. The HA titer of the recombinant virus increased to 2^8–9^ at 72 h and decreased to 2^6–7^ at 96 h. **(E)** Passaged rFLU-HMPV/F-NS (P1~P5) was assessed for HA positivity. **(F)** P1 ~ P5 viral titers of rFLU-HMPV/F-NS were assessed *via* TCID_50_.

To fully characterize rFLU-HMPV/F-NS, firstly, we assessed the expression of HMPV-F and PR8-NP in IAV and rFLU-HMPV/F-NS infected cells by immunofluorescence. Robust IAV-NP staining was detected in both PR8 and rFLU-HMPV/F-NS cells, with no fluorescence observed in uninfected cell controls (Figure 2A). Importantly, rFLU-HMPV/F-NS infection led to the expression of HPMV F protein that was absent in PR8 and uninfected cells, confirming the successful production of this virus (HMPV-F; red staining; Figure 2A). Morphologically, rFLU-HMPV/F-NS virions were spherical and intact when assessed by TEM (Figure 2B) and possessed an identical size distribution to native PR8-IAV (Figure 2C). The rFLU-HMPV/F-NS was diluted 10^−1^ times to infect MDCK cells. The virus titers were measured in supernatant collected from infected cells at 12, 24, 48, 72, and 96 h. The results showed that the HA titer of the rFLU-HMPV/F-NS reached a peak of 2^8–9^ at 72 h, the viral titer started to decrease to 2^6–7^ from 72 to 96 h (Figure 2D). The rFLU-HMPV/F-NS could passage in SPF chicken embryos for five consecutive generations, with a stable haemagglutination potency of 2^7–10^ for generations P1–P5 (Figure 2E) and a TCID_50_ titer of 10^7–9^ TCID_50_/ml (Figure 2F). Together, these results indicated that the rFLU-HMPV/F-NS could perform its function normally and validated the use for future immunization studies.

### Immunogenicity of rFLU-HMPV/F-NS

**Figure 3:**
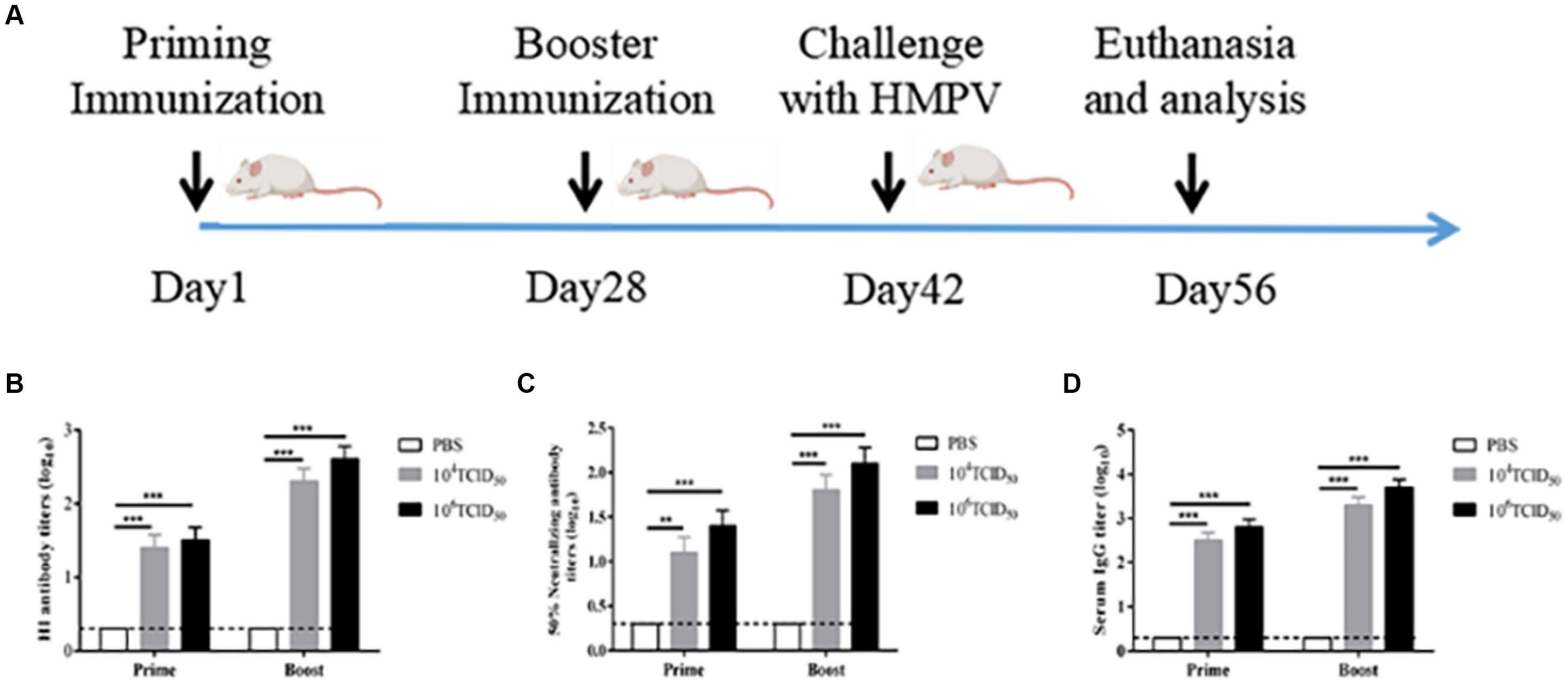
The immunogenicity of recombinant virus rFLU-HMPV/F-NS. **(A)** Schematic outlining the animal immunization schedule. **(B)** HI antibody titers against A/China/BJMY011/2020 (H1N1) in mice immunized with rFLU-HMPV/F-NS. Values are the mean ± SE of three independent experiments (^**^p < 0.01, ^***^p < 0.001). **(C)** Neutralizing antibody titers against HMPV in mice immunized with rFLU-HMPV/F-NS. **(D)** IgG titers against HMPV in mice immunized with rFLU-HMPV/F-NS.

To evaluate the immunogenicity of rFLU-HMPV/F-NS, we immunized BALB/c mice intranasally as shown in Figure 3A. The hemagglutination inhibition assay (HI) was performed to detect anti-influenza virus A/China/BJMY011/2020 (H1N1) in the sera of mice on day 28 after the prime immunization and day 14 after the booster immunization (Figure 3B), both the low-dose and high-dose groups produced highly efficient HI antibodies against influenza virus after prime and booster immunization compared to the PBS control group (*p* < 0.001). It can be tentatively concluded that the rFLU-HMPV/F-NS can effectively induce the production of anti-influenza virus HI antibodies, which is important for mice against influenza virus.

The potency of anti-HMPV neutralizing antibodies was measured after the prime immunization and booster immunization (Figure 3C). Compared with the PBS control group, the low-dose group showed significant neutralizing antibody potency after prime immunization (*p* < 0.01), and both the high-dose group after prime immunization and the low and high-dose groups after booster immunization showed highly significant neutralizing antibody potency (*p* < 0.001). The antibody potency of both the high and low-dose groups after booster immunization was higher than that after prime immunization (*p* < 0.01). We preliminarily assess that rFLU-HMPV/F-NS can effectively induce the production of anti-HMPV neutralizing antibodies and can protect mice from HMPV infection.

### rFLU-HMPV/F-NS effectively induces humoral anti-HMPV immune response

To evaluate the humoral immune responses induced following rFLU-HMPV/F-NS immunization in mice, we measured HMPV-specific IgG antibody expression by ELISA (Figure 3D). The low and high-dose groups after prime and booster immunization produced highly efficient HMPV-specific IgG antibodies, compared with the PBS control group (*p* < 0.001), and the potency of IgG antibodies in both high and low-dose groups after booster immunization was higher than that of prime immunization (p < 0.01). Taken together, the results revealed that the recombinant virus could induce HMPV-specific humoral immune responses in mice.

### rFLU-HMPV/F-NS effectively induces mucosal anti-HMPV immune response

**Figure 4:**
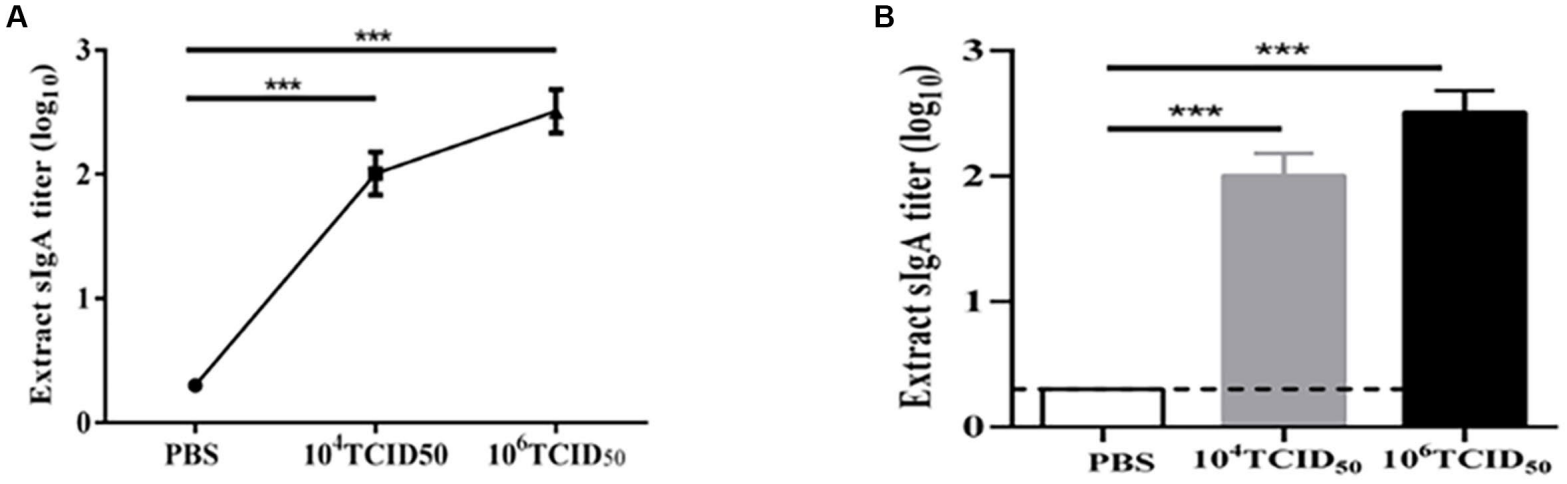
sIgA antibody titers against HMPV in mice immunized with rFLU-HMPV/F-NS. **(A)** Line bars and **(B)** graphical representation of HMPV-induced sIgA values calculated from the serum samples of immunized mice by ELISA plates. Values are the mean ± SE of three independent experiments (^***^p < 0.001).

To determine the mucosal immune response stimulated by the recombinant virus, we measured HMPV-specific sIgA antibody expression by ELISA (Figure 4). The levels of HMPV-specific sIgA antibodies secreted in the lung lavage fluid of mice in the low-dose and high-dose groups were extremely significantly higher compared with the PBS control group (*p* < 0.001). The potency of HMPV-specific sIgA antibodies in the high-dose group was higher than in the low-dose group (*p* < 0.05). It suggests that recombinant virus rFLU-HMPV/F-NS can induce mucosal immune responses in the organism.

### Cellular immune response *in vivo*

**Figure 5:**
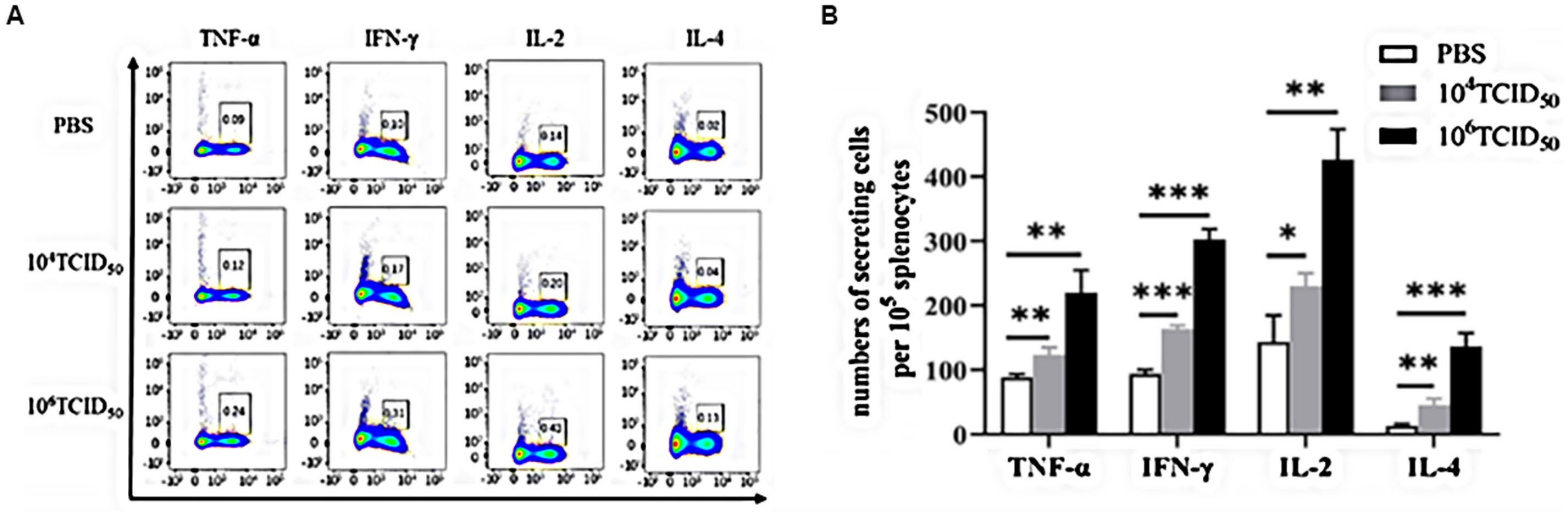
The level of cytokines in mice immunized with rFLU-HMPV/F-NS. Cytokine expression of HMPV-specific Th1/Th2 cytokines (TNF-α, IFN-γ, IL-2, and IL-4) in mouse spleen lymphocytes were assessed by flow cytometry. **(A)** Representative flow cytometric gating. **(B)** Graphical representation of the number of cells per 10^5^ solenocytes Values are the mean ± SE of three independent experiments; ^*^p < 0.05, ^**^p < 0.01, ^***^p < 0.001.

We verified Th1/Th2 type immune response induced by rFLU-HMPV/F-NS *via* flow cytometry analysis(Figure 5). The low dose group induced a highly significant increase in the HMPV-specific cytokines TNF-α(Th1-type), IL-4(Th2-type; *p* < 0.01) and IFN-γ(Th1-type; *p* < 0.001) and a significant increase in the IL-2(Th1-type; *p* < 0.05), and the high dose group induced a highly significant increase in the HMPV-specific cytokines TNF-α, IL-2(Th1-type; *p* < 0.01), IFN-γ(Th1-type) and IL-4(Th2-type; *p* < 0.001) were highly significantly elevated. The results allow for the induction of a Thl/Th2 immune response by recombinant virus rFLU-HMPV/F-NS, with a tendency toward a Th1-type cellular immune response. This provides data on the balance of Thl/Th2 immune response in recombinant HMPV vaccines.

### Immune protection against the wild-type HMPV challenge following immunization with rFLU-HMPV/F-NS

**Figure 6:**
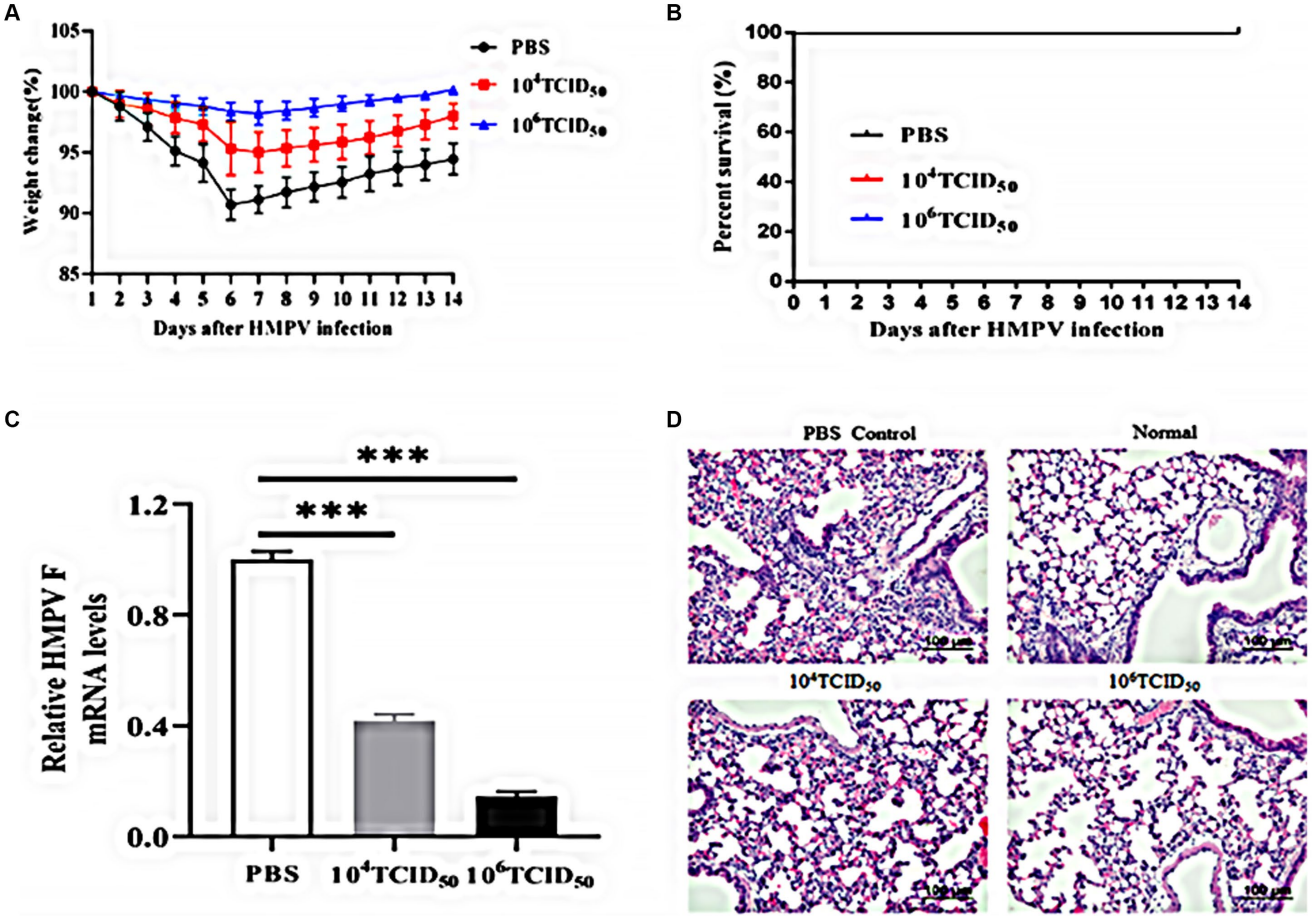
Immunoprotective effects of rFLU-HMPV/F-NS following HMPV challenge. **(A)** Weight changes; **(B)** Survival curves; **(C)** HMPV viral load (^***^p < 0.001) and **(D)** Histopathology (×200) in the lung of immunization mice against HMPV.

We assessed the effects of HMPV challenge on mice immunized with rFLU-HMPV/F-NS by body weight change and survival rate. The body weight of the mice in the high dose group did not change significantly (Figure 6A), and no mortality in mice after HMPV infection (Figure 6B).

To further evaluate whether the rFLU-HMPV/F-NS can protect mice from HMPV attack, we measured the HMPV viral load and pathological changes in the lung tissues of mice. The low-dose and high-dose groups could effectively inhibit the replication of HMPV in lung tissues, with the high-dose group showing a more pronounced inhibition(*p* < 0.001; Figure 6C). Compared with the lung sections of normal mice, the lung tissue of the PBS group showed alveolar fusion and a large number of lymphocytes were observed under the microscope. Lung histopathological damage was less severe and lymphocyte infiltration was reduced in the low-dose and high-dose groups compared with the PBS group (Figure 6D). Thus, recombinant virus rFLU-HMPV/F-NS had a significant immunoprotective effect against HMPV infection, and the high-dose group exhibited more effective immunoprotection.

### Immune protection against the wild-type influenza virus challenge following immunization with rFLU-HMPV/F-NS

**Figure 7:**
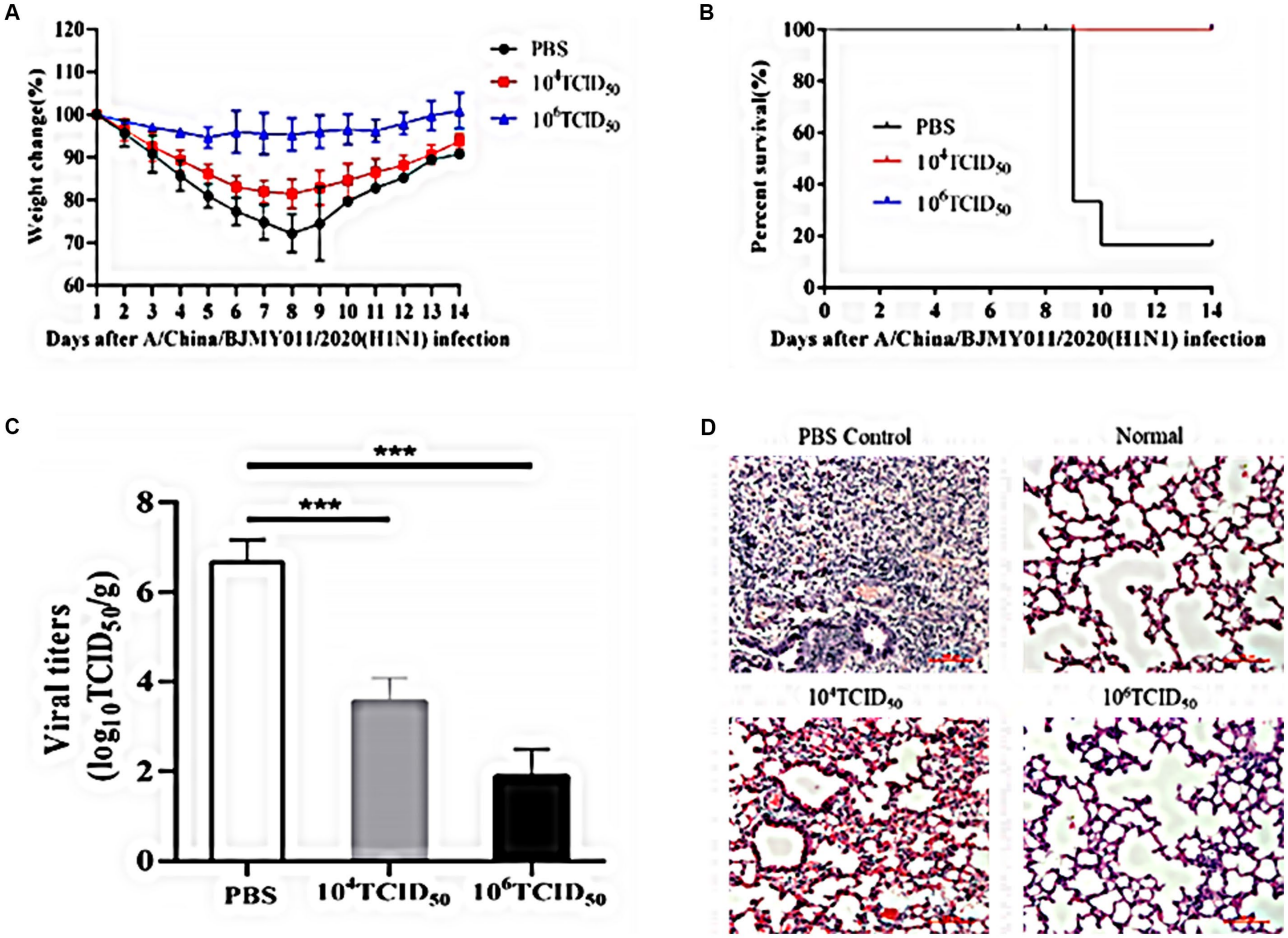
Immunoprotective effects of rFLU-HMPV/F-NS following A/China/BJMY011/2020 (H1N1) challenge. **(A)** Weight changes; **(B)** Survival curves; **(C)** Viral titers (^***^p < 0.001) and **(D)** Histopathology (×200) in the lung of immunization mice against A/China/BJMY011/2020 (H1N1).

To evaluate the protective effect exerted by rFLU-HMPV/F-NS immunized mice against influenza virus challenge, we assessed the body weight and survival rate of mice immunized with rFLU-HMPV/F-NS for 14 days. The body weight of the mice in the high-dose group did not change significantly (Figure 7A), and no mortality in the immunized group after infection with influenza A/China/BJMY011/2020 (H1N1) virus, while the PBS control group showed substantial mortality at day 9 (Figure 7B). It was tentatively shown that the recombinant virus rFLU-HMPV/F-NS could protect mice from the challenge of influenza A/China/BJMY011/2020 (H1N1) virus.

To further evaluate the protective effect of rFLU-HMPV/F-NS in mice, we measured the TCID_50_ titer and pathological changes in mouse lung tissues of influenza virus. Both the low-dose and high-dose groups effectively inhibited the replication of influenza virus in lung tissues, with the inhibition in the high-dose group being more pronounced (*p* < 0.001; Figure 7C).

Lung tissue in the PBS group had a large number of lymphocyte infiltrates compared to lung sections from normal mice. Lung histopathological damage was reduced and lymphocyte infiltration was decreased in the low-dose and high-dose groups compared with the PBS group (Figure 7D).

Thus, recombinant virus rFLU-HMPV/F-NS showed significant immunoprotection against A/China/BJMY011/2020 (H1N1) infection, and the immunoprotective effect in the high-dose group was more in line with our expectation.

## Discussion

HMPV infection presents a worldwide disease burden without a commercial vaccine against it. Recombinant viral vaccine candidates using influenza viruses as vectors have played an integral role in preventing many infectious diseases and in cancer control. The smallest fragment of influenza virus, NS, encodes two proteins, NS1 and NEP, a multifunctional nuclear export protein involved in mediating the export of vRNPs from the host nucleus (Baranovskaya et al., 2019). The NS1 protein is involved in a variety of viral-host interaction processes and its primary function is to suppress the cellular immune response to viral infection. A genetically engineered deletion in the NS1 open reading frame results in viral attenuation (Hale et al., 2008; Baranovskaya et al., 2019). In this study, we developed a novel recombinant influenza virus, rFLU-HMPV/F-NS, in which the HMPV F coding gene was cloned into the PR8 non-structural protein NS1, termed rFLU-HMPV/F-NS. The morphological structure and size distribution of rFLU-HMPV/F-NS particles were consistent with those of IAV as observed by electron microscopy. rFLU-HMPV/F-NS also successfully expressed HMPV F and PR8 NP and could be stably transmitted in cell culture.

Several studies have reported that many HMPV vaccines exhibit immune damage problems, such as enhanced disease and delayed viral clearance due to excessive activation of Th2-type immune responses in animal experiments(Schickli et al., 2009; Anderson, 2013; Aerts et al., 2015). In our study, we evaluated recombinant virus-induced immune response phenotypes by some popular representative factors of Th1-type and Th2-type immune responses(Dubois et al., 2019; Stepanova et al., 2020). rFLU-HMPV/F-NS activated Th1/Th2 type immune responses *in vivo* and favored Th1 type cellular immune responses, inducing a Th1/Th2 type balance. And when mice were challenged with HMPV 14 days after the booster immunization, no significant body weight fluctuations were observed, lung tissue lesions in immunized mice were reduced and HMPV viral load was significantly decreased, data that emphasize the effective immunoprotective effect it produces against HMPV.

The mechanisms by which HMPV regulates host immunity are being intensively investigated, but many questions remain unanswered. An effective HMPV vaccine would ensure the induction of a prompt and appropriate immune response (generation of immune memory) following natural HMPV infection of the host (Herd et al., 2006; Ren et al., 2015). Accurate stimulation of CD4 T cells to increase antibody production following vaccine immunization and the formation of a rapid and effective secondary CTL response of CD8 memory T cells to natural HMPV infection are key issues in the study of effective HMPV vaccines (Hamelin et al., 2007; Kolli et al., 2008; González et al., 2017; Stepanova et al., 2020). Therefore, studying the CD4 branch of HMPV immunity is a key requirement for controlling the pathological process of HMPV and developing prevention strategies. Therefore, our subsequent studies need to explore the CD4 branch of HMPV immunity in depth, which will help us to understand the pathological process of HMPV more clearly and develop prevention strategies.

The success of our rFLU-HMPV/F-NS vaccine in protecting mice from HMPV attack and influenza virus attack is prominent. These new data support more in-depth exploration and development of HMPV vaccines against recombinant influenza viruses, such as optimizing vaccine doses, assessing whether other methods of vaccine administration such as pulmonary inhalation may have an impact on their immunoprotective effects, and evaluating the efficacy, stability and safety of rFLU-HMPV/F-NS recombinant viruses in other experimental animals such as cotton mice and primates, which are necessary for providing theoretical assurance for the screening of HMPV vaccine strains to protect children from the disease consequences of HMPV infection, and to provide ideas for “one vaccine for two uses.”

## Data availability statement

The original contributions presented in the study are included in the article/supplementary material, further inquiries can be directed to the corresponding author.

## Ethics statement

The animal study was reviewed and approved by the Ethics Committee of the Fifth Medical Center of Chinese PLA General Hospital.

## Author contributions

TC: conceptualization, methodology, software, investigation, formal analysis, and writing—original draft. LG: data curation, investigation, formal analysis, and writing—original draft. SF: visualization and investigation. DZ: resources and supervision. YH: software and validation. LC: visualization and supervision. LX: resources and software. HW: visualization and validation. TL: software and supervision. NY: validation and supervision. YP: conceptualization, funding acquisition, resources, supervision, and writing—review. All authors contributed to the article and approved the submitted version.

## Funding

Sources of funding for this work include National Key Research and Development Program of China (2019SWAQ05-3-5) and National Natural Science Foundation of China (81971564 and 8200010324).

## Conflict of interest

The authors declare that the research was conducted in the absence of any commercial or financial relationships that could be construed as a potential conflict of interest.

## Publisher’s note

All claims expressed in this article are solely those of the authors and do not necessarily represent those of their affiliated organizations, or those of the publisher, the editors and the reviewers. Any product that may be evaluated in this article, or claim that may be made by its manufacturer, is not guaranteed or endorsed by the publisher.
